# Skeletal Muscle Inflammation Following Repeated Bouts of Lengthening Contractions in Humans

**DOI:** 10.3389/fphys.2015.00424

**Published:** 2016-01-12

**Authors:** Michael R. Deyhle, Amanda M. Gier, Kaitlyn C. Evans, Dennis L. Eggett, W. Bradley Nelson, Allen C. Parcell, Robert D. Hyldahl

**Affiliations:** ^1^Department of Exercise Sciences, Brigham Young UniversityProvo, UT, USA; ^2^Department of Statistics, Brigham Young UniversityProvo, UT, USA; ^3^Department of Natural Sciences, Ohio Dominican UniversityColumbus, OH, USA

**Keywords:** IP-10, CXCL10, MCP-1, CCL2, eccentric exercise, damage, T-cell

## Abstract

Skeletal muscle responds to exercise-induced damage by orchestrating an adaptive process that protects the muscle from damage by subsequent bouts of exercise, a phenomenon called the repeated bout effect (RBE). The mechanisms underlying the RBE are not understood. We hypothesized that an attenuated inflammation response following a repeated bout of lengthening contractions (LC) would be coincidental with a RBE, suggesting a potential relationship. Fourteen men (*n* = 7) and women (*n* = 7) completed two bouts of lengthening contractions (LC) separated by 28 days. Muscle biopsies were taken before the first bout (B1) from the non-exercised leg, and from the exercised leg 2- and 27-d post-B1 and 2-d following the second bout (B2). A 29-plex cytokine array identified alterations in inflammatory cytokines. Immunohistochemistry quantified inflammatory cell infiltration and major histocompatibility complex class 1 (MHC-1). Muscle soreness was attenuated in the days following B2 relative to B1, indicating a RBE. Intramuscular monocyte chemoattractant protein (MCP1) and interferon gamma-induced protein 10 (IP10) increased following B2 relative to the pre-exercise sample (7–52 and 11–36 pg/ml, respectively *p* < 0.05). Interleukin 4 (IL4) decreased (26–13 pg/ml, *p* < 0.05) following B2 relative to the pre-exercise sample. Infiltration of CD68^+^ macrophages and CD8^+^ T-cells were evident following B2, but not B1. Moreover, CD8^+^ T-cells were observed infiltrating apparently necrotic muscle fibers. No changes in MHC-1 were found. We conclude that inflammation is not attenuated following a repeated bout of LC and that CD8^+^ T-cells may play a role in muscle adaptation following LC. Moreover, it appears that the muscle or the immune system becomes sensitized to an initial bout of damaging exercise such that inflammatory cell infiltration into the muscle is enhanced upon a repeated bout of damaging exercise.

## Introduction

A truly remarkable property of skeletal muscle is that it maintains an intrinsic protective mechanism, whereby it swiftly adapts following exercise-induced damage, making it capable of resisting future damage. This phenomenon has been recognized for over half a century (Highman and Altland, 1963) and is commonly referred to in the literature as the repeated bout effect (RBE) (Nosaka and Clarkson, 1995). Over the years, a significant body of literature has been developed to describe the parameters of an original stimulus necessary to induce the protective effect (Lavender and Nosaka, 2008; Barroso et al., 2010; Muthalib et al., 2011). Nevertheless, the molecular underpinnings and deeper mechanisms of the RBE have been relatively understudied and are not well known. A more complete understanding of the mechanisms that mediate the RBE adaptation(s) may have significant clinical value with vast applications, from the prevention and management of muscle related injuries, to the treatment of muscle degenerative disorders.

Potential mechanisms for the RBE that have been explored recently by our laboratory and others have implicated extracellular matrix remodeling (Hyldahl et al., 2015), and muscle architectural alterations (Lau et al., 2015). An older hypothesis, supported initially by the results of animal studies, proposes that reduced inflammation following a second damage exposure reduces secondary damage and subsequent markers of muscle damage (McHugh, 2003). Indeed rodent studies have shown a blunted inflammatory response in conjunction with muscle damage markers when lengthening contractions (LC) were preceded by either a bout of passive stretches, or a prior bout of LC (Pizza et al., 2002; Koh et al., 2003). However, inflammation has not been comprehensively assessed following repeated bouts of damaging exercise in humans, leaving doubt as to whether this mechanism is important in mediating the protective effect in human skeletal muscle. Moreover, few studies have examined the intramuscular inflammatory cytokine environment following repeated bouts of damaging exercise. Information from these studies is also somewhat limiting given that the cytokines were measured only at the mRNA level (Hubal et al., 2008; Mackey et al., 2011).

Thus, the purpose of this study was to broadly assess markers of inflammation (i.e., cytokines and infiltrating lymphoid and myeloid cells) in human muscle in a well-established paradigm of the RBE in the knee extensor muscles. Overall, we hypothesized that muscle inflammatory markers would be present following the first bout of LC, as reported by others (Paulsen et al., 2012). Furthermore, we expected that these same markers would be attenuated following a repeated bout of LC, consistent with the attenuation of other direct and indirect markers of muscle damage.

## Methods

### Subjects

Fourteen young, healthy, men (*n* = 7; age 23.3 ± 2.1) and women (*n* = 7; age 25.6 ± 2.5) volunteered to participate in this study. The activity level of the subjects ranged from moderately active to sedentary. The subjects had not participated in weight training or consistent, structured physical activity in the past 6 months. As a participant in the study, the subjects agreed to not change their activity levels or use any form of analgesic or non-steroidal anti-inflammatory drugs during the duration of the study. All subjects signed a written informed consent document authorized by the Brigham Young University Institutional Review Board, and were informed of the procedures as well as potential risks.

### Study design

This study consisted of two bouts of lengthening contractions (LC) spaced 28 days apart. Twenty eight days between bouts was chosen because it allows for sufficient washout of acute symptoms of the initial bout (e.g., soreness, strength loss, serum enzymes) and is still within the 6 to 9-month window of time that the RBE lasts after an initial bout (Nosaka et al., 2001). Each bout of exercise was performed on the same randomly selected leg. Four muscle biopsies were taken. The first biopsy was taken 1 day before the first bout of exercise (designated as day -1) from the non-exercised leg. Two days after the first bout of exercise, the second biopsy was taken (designated as 2d post-B1) from the exercised leg. The third biopsy (designated as 27d post-B1) was taken from the same exercised leg 27 days after the B1 (also described as the day before the second bout of exercise). The next day (28 days after B1) the subjects completed the second bout of LC (B2) using the same exercise leg, and a muscle biopsy was taken 2 days later on the exercised leg (designated as 2d post-B2). The post-LC biopsy was taken at 48 h because this is around the time when many markers of muscle damage are greatest (strength loss, soreness, plasma creatine kinase activity). Muscle soreness was assessed 1 day prior to the exercise and then again 2, 3, 4, and 5 days following each bout of LC. Throughout the duration of the study, subjects were instructed to maintain their regular diet and level of physical activity, as well as refraining from use of caffeine, alcohol and NSAIDs. One subject reported using ice 2 days after the first bout of exercise to reduce muscle soreness.

### Exercise protocol

The exercise sessions each included 30 sets of 10 maximal eccentric contractions. The exercises were performed on an isokinetic dynamometer (Biodex Medical Systems, Shirley, NY, USA). Subjects were instructed to resist the lever arm from 30° (0° = full extension) of flexion to the subject's maximal knee flexion ROM (approximately 110°), moving at an angular velocity of 120° s^−1^. Between each set, subjects were given a 1-min rest. This same exercise protocol has been widely used to induce muscle damage and a RBE (McKay et al., 2010; Hyldahl et al., 2015). There were no differences in the amount of work performed for each bout of exercise.

### Muscle biopsy procedure

Four total muscle biopsies were taken from the *m. vastus lateralis*. This muscle was chosen because it is easy to access and no major blood vessels or nerves are in the area. After using 2% lidocaine as a local anesthetic, a small incision was made past skin and fascia. Percutaneous muscle biopsies were then collected through a manual suction method. The first biopsy incision was made approximately 15 cm proximal to the insertion of the *vastus lateralis* of the non-exercised leg. Subsequent biopsies from the exercised leg were taken approximately 3–5 cm proximal to the previous biopsy to minimize the effects of previous biopsies. Care was also taken to angle the insertion of the needle away from the previous biopsy to minimize effects of previous biopsies (Van Thienen et al., 2014). Muscle samples were preserved for both immunohisochemisty and protein analysis. Samples for microscopic analysis were mounted on a cork and frozen in isopentane-cooled liquid nitrogen while samples used for protein homogenates were snap frozen in liquid nitrogen. All samples were stored at −80°C.

### Soreness assessments

Perceived muscle soreness was assessed using a visual analog scale (VAS). The VAS consisted of a 100 mm line with anchors indicating “no pain” on one end, and “unbearable pain” on the opposite end. Subjects were instructed to perform two body weight squats and then immediately evaluate soreness by marking on the VAS scale with a vertical line. The distance from the no pain end of the VAS was measured and used for analysis.

### Immunohistochemistry

Muscle biopsy samples used for histochemical staining were cut into 8 μm sections using a cryostat at −25°C. Cut samples were mounted on Superfrost slides and allowed to air dry for 10 min. Sections stained for CD8 were fixed in 2% paraformaldehyde for 10 min. After fixation, samples were washed in phosphate-buffered saline (PBS) and then incubated in primary antibodies overnight at 4°C (CD8 diluted 1:50 in PBS and dystrophin 1:500). On the following day, samples were washed and incubated in secondary antibodies diluted 1:100 in PBS for 30 min at 37°C. DAPI (4′,6-Diamidino-2-Phenylindole, Dihydrochloride) was added to the secondary antibody solution to visualize nuclei. Following incubation, samples were washed in PBS and dipped in water. The slides were then dried and mounted with Fluoroshield histology mounting medium (Sigma-Aldrich, St Louis, Mo, USA). Sections stained for CD68 were fixed in 2% paraformaldehyde for 4–8 min. Following fixation, sections were permeabilized in 0.2% Triton X-100 for 10 min and then blocked in a 2% bovine serum albumin (BSA), 5% fetal bovine serum (FBS) solution for 30 min at room temperature. Sections were incubated in the primary antibodies (CD68 and dystrophin diluted in blocking cocktail at 1:300 and 1:200, respectively) in a humidified chamber overnight at 4°C. Following several washes, sections were then incubated in the appropriate secondary antibodies (both diluted at 1:200 in PBS) for 30 min at 37°C. Following multiple washes, slides were incubated in DAPI for 30 min at 37°C. Stained slides were washed in PBST (PBS tween-20), dried, then mounted using Fluoroshield histology mounting medium. Sections stained for MHC-I were fixed in 100% ice-cold acetone for 10 min. Following a rinse with PBS, the samples were placed in a humidified chamber and were incubated in blocking solution for 1 h (5% horse serum and 0.2% Trition X 100). Following blocking, the samples were washed in PBS. The samples were then incubated with the primary antibody diluted 1:100 in PBS with 2% BSA in a humidified chamber for 1 h at room temperature. The wash procedure was repeated and the samples were incubated in a secondary antibody diluted 1:100 in PBS with DAPI. All slides were imaged on an Olympus IX73 fluorescence capable inverted microscope. Antibody stain validity was verified using secondary-only controls. Primary antibodies used were; CD8, mouse monoclonal IS623 (Dako, Glostrup, Denmark), CD68, mouse monoclonal M0718 (Dako, Glostrup, Denmark), dystrophin, rabbit polyclonal (ab15277; Abcam, Cambridge, United Kingdom) HLA-ABC (MHC-I) mouse monoclonal M0736 (Dako, Glostrup, Denmark). Secondary antibodies used: Alexa Fluor 488 goat anti-rabbit A11029 (Life Technologies, Carlsbad California, USA), Cy3 anti-rabbit 115-165-003 (Jackson ImmunoResearch Laboratories, West Grove PA).

### Quantification of immunofluorescent images

Quantification of immunofluorescent images was carried out by investigators that were blind to both condition and time point. For CD68 analyses, enumeration was accomplished by analyzing 10 randomly acquired fields using a 40X objective. A cell was considered CD68^+^ when the green fluorescent stain clearly surrounded a DAPI^+^ nucleus (see arrows in **Figure 4**). An average of 210 ± 10 muscle fibers were analyzed per subject per time point. Because CD8^+^ cells appeared to be much less frequent than CD68^+^ cells, CD8 enumeration was made by imaging the entire section, using a 20X objective. This resulted in approximately 10–20 acquired fields per time point, depending on the size of the muscle cross section. An average of 801.9 ± 329.3 muscle fibers were analyzed per subject per time point. To assess MHC-I content, the entire muscle section was imaged at 10 X magnification. Two to five images per time point were acquired to capture the entire section. Images were taken using the same exposure and gain settings within subjects. For quantification, the total MHC-1 immunoreactive area was expressed relative to the total area of the imaged section.

### Cytokine magnetic bead multiplex

The frozen muscle samples were homogenized with a Total Protein Extraction Kit (Millipore, Billerica, MA) with protease and phosphatase inhibitor cocktails (Thermo Scientific, Rockford, IL). A Direct Detect Spectrometer (EMD Millipore, Billerica, MA) was used to determine the total protein concentrations. The Luminex Magpix multiplexing platform was then used for multianalyte profiling of biopsy sample homogenates. Cytokines in muscle homogenates were measured using a 29-plex cytokine kit in compliance with manufacturer's parameters (EDM Millipore, Billerica, MA). Briefly, 25 μg of protein homogenate were incubated overnight at 4°C with antibody-conjugated magnetic beads. The bead-complex was then washed, followed by 30 min incubation at RT on a plate shaker in biotinylated detection antibody. Streptavidin-phycoerythrin was subsequently added and samples were incubated for an additional for 30 min on a plate shaker at RT. A Magpix (Luminex Corporation, Austin, TX) system was used to quantify bead-complexes. Data analysis was based on a minimum of 80 beads using median fluorescence values.

### Statistics

Perceived muscle soreness was evaluated using the area under the curve (AUC) for soreness response for the days after bout 1 and bout 2 for each subject. AUC measurements 1 day before exercise and days 2 through 5 after exercise were used to calculate AUC for each bout. A paired *t*-test was used to test for differences in AUC between bouts. A One-way repeated measures analysis of variance (ANOVA) was used to test for differences in immunohistochemical data. Each individual cytokine of interest was also analyzed using One-way repeated measures ANOVAs. A Tukey-Kramer HSD test was used for pairwise comparisons when the *F*-statistic revealed a significant *p*-value. Correlation analyses were carried out using a mixed models linear regression technique with blocking on subjects. Blocking on subjects was used to account for the lack of independence in the data. Correlation analysis was only investigated when a relationship between variables was anticipated. To normalize distributions and homogenize variance, the following variables were log transformed; IL-4, MCP-1, IP-10, soreness. Prism Graphpad (V6.0b; San Diego, CA, USA) was used for AUC and *t*-test calculations for soreness data. JMP® Pro (V11.2; SAS institute, Cary, NC, USA) was used for all other analyses. Results were considered statistically significant at *p* < 0.05.

## Results

### Soreness

**Figure 1:**
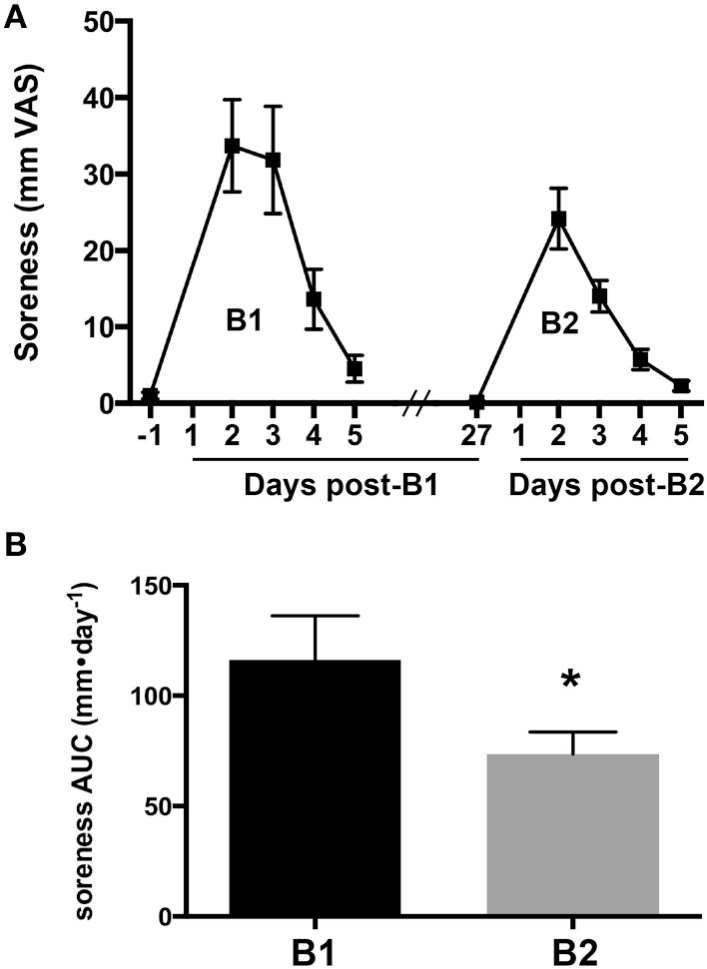
**Delayed onset muscle soreness as an indirect marker of muscle damage**. **(A)** Soreness response curves measured on a visual analog scale (VAS) for 1 day before and 5 days following the first (B1) and second bouts (B2) of lengthening contractions (LC). **(B)** The area under the curve (AUC) for muscle soreness for B1 and B2. ^*^ Indicates significant difference (paired *t*-test, *p* < 0.05).

Changes in the overall magnitude of delayed onset muscle soreness associated with B1 and B2 were compared to verify the presence of a RBE. Muscle soreness in the knee extensor muscle group increased after B1 and B2 and returned to baseline values 5 days after exercise for both bouts (Figure 1A). Muscle soreness peaked 2 days after exercise for B1 and B2. To quantify differences in overall soreness response in the days after each bout, we compared the AUC of soreness for B1 and B2. AUC of soreness was significantly reduced at B2 compared to B1 (33% reduction, ± 11%, one-tailed *p* = 0.032; Figure 1B).

### Cytokine array

**Figure 2:**
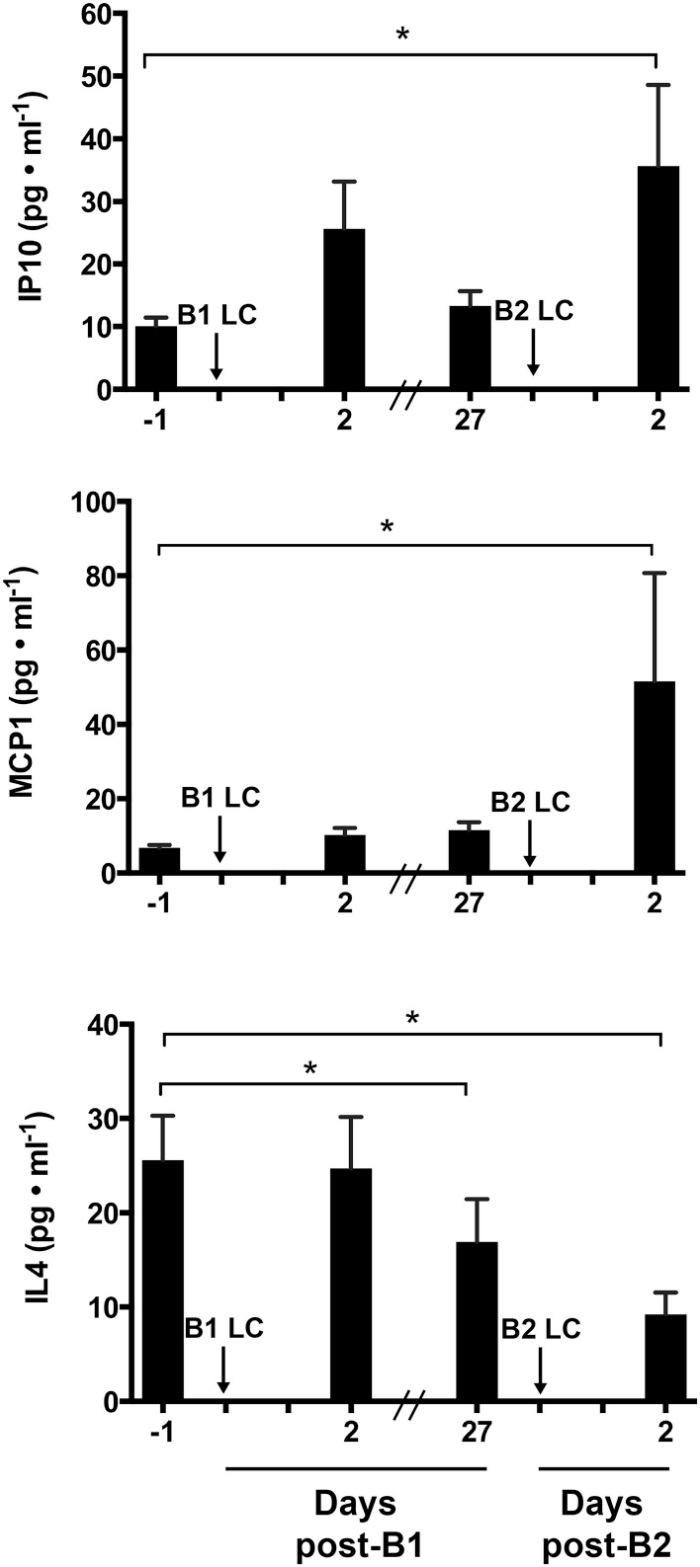
**Intramuscular cytokine protein concentration before and 2 days following the first (B1) and second bouts (B2) of lengthening contractions (LC)**. Abbreviations: IP-10, Interferon gamma-inducible protein 10; MCP-1, Monocyte chemoattractant protein 1; IL-4, interleukin 4. ^*^ Indicates significant differences between two samples (One-way repeated measures ANOVA with Tukey's HSD, *p* < 0.05).

To determine the extent of inflammation and identify potentially important inflammatory cytokines that may mediate the RBE, we performed an unbiased 29-plex cytokine screen. Among the 29 cytokines measured, only monocyte chemoattractant protein 1 (MCP-1 also known as CCl2), Interleukin 4 (IL-4), and Interferon-gamma-inducible protein 10 (IP-10 also known as CXCL10) showed a significant main effect among biopsy time points (*p* < 0.05). Figure 2 shows comparisons of the mean values of IP-10, MCP-1, and IL-4 across sampling time points. IP-10 was significantly increased at post-B2 relative to pre-B1 (2.26 ± 1.31-fold, *p* = 0.021). Potential trending differences may be present between pre-B1 and post-B1 (*p* = 0.11) and pre-B2 and post-B2 (*p* = 0.12). MCP-1 content at post-B2 was significantly elevated compared to pre-B1 (2.97 ± 1.35-fold, *p* = 0.0032). MCP-1 content at pre-B2 showed a suggestive but inconclusive increase over pre-B1 (2.15 ± 1.35-fold *p* = 0.0603). In contrast, IL-4 showed a significant decrease from pre-B1 to post-B2 (3.82 ± 1.44-fold, *p* = 0.041) and from pre-B1 to pre-B2 (3.72 ± 1.44-fold, *p* = 0.005). An inconclusive decreasing trend in IL-4 content was also observed between pre-B2 and post-B2 (*p* = 0.14) Results of all other non-significant detectable cytokines can be found in Table 1.

**Table 1:** **Cytokine concentrations from skeletal muscle biopsy samples 24 h before (Pre) and 48 h after (Post) two bouts of exercise consisting of 300 maximal lengthening contractions (LC) separated by 27 days**.

**Cytokine**	**Abbreviation**	**Bout 1 LC**		**Bout 2 LC**	
		**Pre (pg ml^−1^)**	**48 h Post (pg ml^−1^)**	**Pre (pg ml^−1^)**	**48 h Post (pg ml^−1^)**
Interferon gamma-inducible protein 10	IP-10	10.1±1.3	25.6±7.6	13.3±2.4	35.7^*^±12.9
Monocyte chemoattractant protein 1	MCP-1	6.8±0.7	10.3±1.9	11.5±1.14	51.6^*^±29.1
Interleukin 4	IL-4	25.6±4.7	24.7±5.5	18.1^*^±4.5	12.5^*^±2.1
Granulocyte colony-stimulating factor	GCSF	23.8±5.3	32.1±4.5	32.2±4.5	27.2±4.3
Interferon alpha 2	IFNa-2	34.5±3.1	35.3±3.0	33.9±2.6	31.3±2.1
Interleukin 13	IL-13	5.9±0.8	6.6±0.7	5.9±0.5	5.7±0.5
Interleukin 5	IL-5	2.9±0.2	2.8+0.2	2.8±0.2	2.4±0.1
Interleukin 6	IL-6	13.0±1.6	2.8±1.7	13.0±1.6	11.0±1.3
Interleukin 7	IL-7	2.5±0.1	2.4±0.1	2.4±0.2	2.4±0.1
Vascular endothelial growth factor	VEGF	25.8±2.0	31.3±3.6	27.9±2.5	26.3±3.9

*Data are means ± SEM of 10 detectible cytokines. Cytokines were deemed undetectable when the majority (>50%) of the observations were below the minimum detection limit. The minimum detection limit ranged from 0.82 to 2.18 pg/ml*.

*The 19 undetectable cytokines were: EGF, epidermal growth factor; Eotaxin; GMCSF, granulocyte-macrophage colony-stimulating factor; IFNy, interferon gamma; IL-10, interleukin 10; IL-12p40, interleukin 12 p40; IL-12p70, interleukin 12 p70; IL-15, interleukin 15; IL-17A, interleukin 17; IL-1 RA, alpha interleukin 1 receptor antagonist; IL-1a, interleukin 1 alpha; IL-1b, interleukin 1 beta; IL-2, interleukin 2; IL-3, interleukin 3; IL-8, interleukin 8; MIP-1a, macrophage inflammatory protein 1 alpha; MIP-1b, macrophage inflammatory protein 1 beta; TNF-a, tumor necrosis factor alpha; TNF-b, tumor necrosis factor b*.

* *Indicates statistically significant difference from pre Bout 1 LC (One-way repeated measures ANOVA with Tukey's HSD, p < 0.05)*.

### Macrophage infiltration

**Figure 3:**
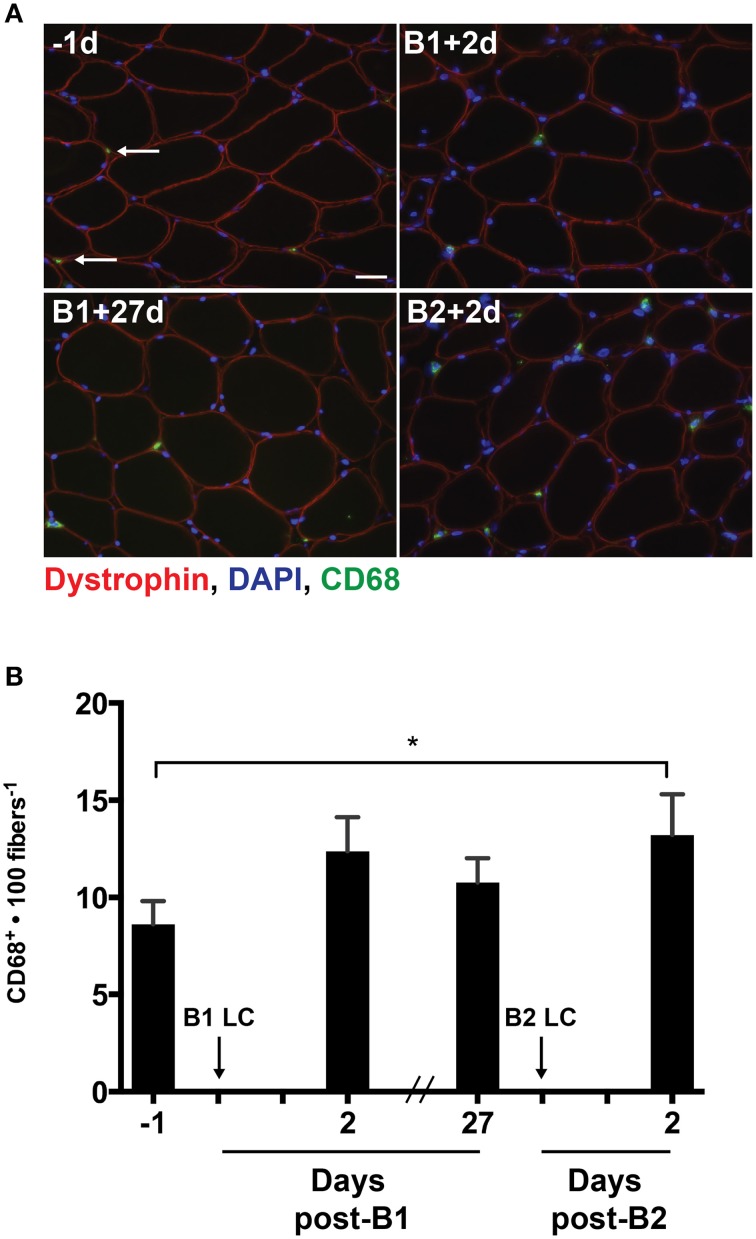
**Muscle-infiltrating CD68^+^ macrophages**. **(A)** Eight micrometer thick muscle cross-section stained for dystrophin (red), CD68 (green), and DNA (blue). All four images were taken from muscle samples from the same subject 1 day before bout 1 (B1) of damaging lengthening contractions (LC) (-1d), 2 days after B1 (B1 + 2d), 27 days after bout 1 (B1 + 27d) and 2 days after bout 2 (B2) (B2 + 2d). Arrows indicate a CD68^+^ macrophage as evidenced by CD68 immunoreactivity surrounding a blue nucleus. **(B)** Intramuscular CD68^+^ macrophage enumeration per muscle fiber before and 2 days following the first (B1) and second bout (B2) of damaging lengthening contractions (LC). ^*^ Indicates significant difference (One-way repeated measures ANOVA with Tukey's HSD, *p* < 0.05). Scale bar = 20 μm.

As MCP-1 is an important mediator of macrophage infiltration into damaged skeletal muscle (Shireman et al., 2007), we analyzed the muscle cross sections for the presence of CD68^+^ macrophages using immunohistochemistry (Figure 3A). Given that a significant increase in MCP-1 was only detectable following B2, we expected that macrophage content would be increased post-B2 relative to the pre-B1 measurement. As expected, the number of CD68^+^ cells per 100 muscle fibers increased by 4.6 ± 1.5 cells per 100 fibers following B2 compared to the pre-B1 measurement (*p* = 0.021; Figure 3B). Additionally we noted an increasing trend in the number of CD68^+^ macrophages at post-B1 compared to the pre-B1 measurement (*p* = 0.08).

### T-cell infiltration

**Figure 4:**
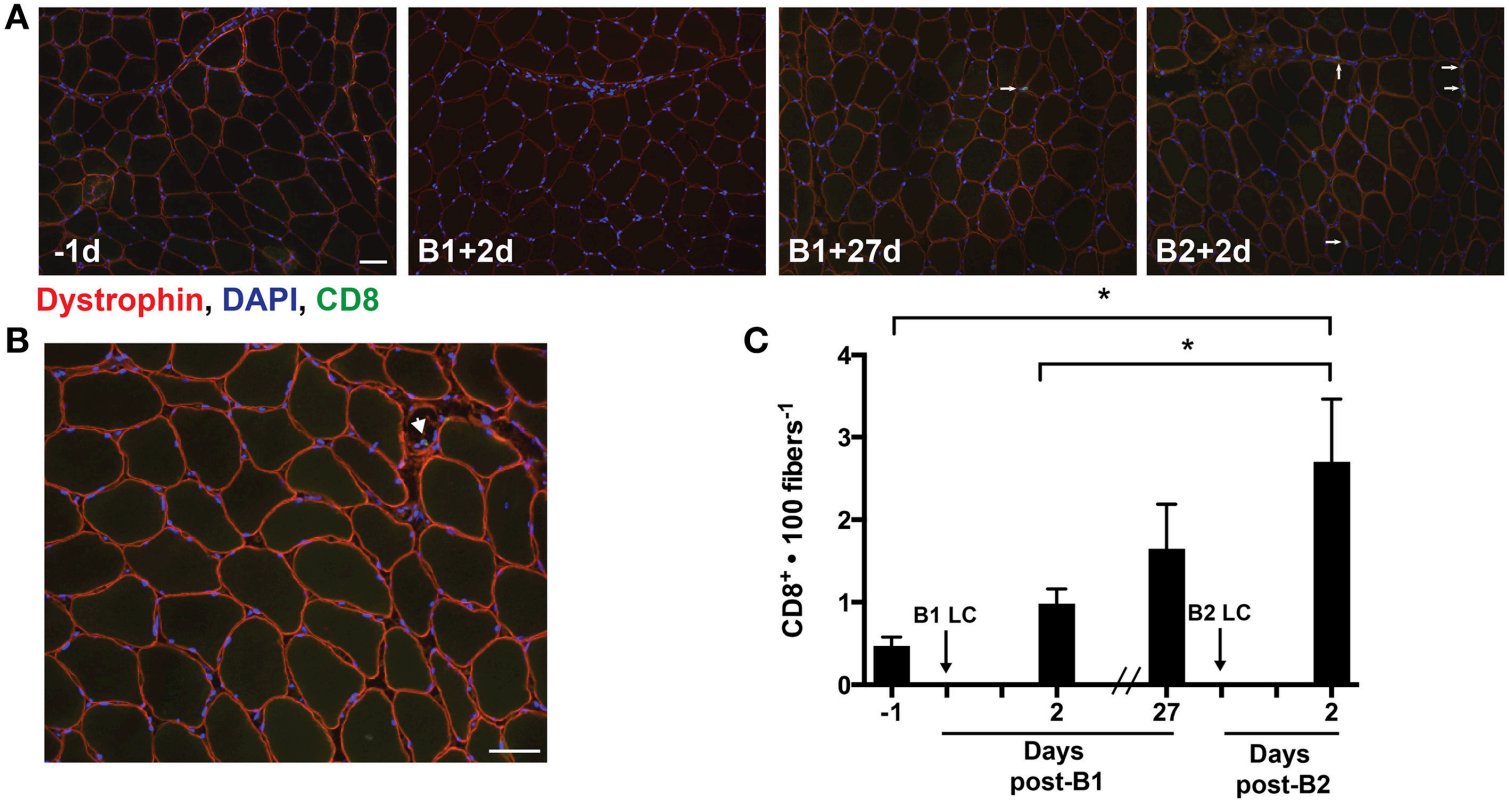
**Muscle-infiltrating CD8^+^ T-cells**. **(A)** Eight micrometer thick sections stained for dystrophin (red), DNA (blue), and CD8 (green). All four images were taken from muscle samples 1 day before bout 1 (B1) of damaging lengthening contractions (LC) (−1d), 2 days after B1 (B1 + 2d), 27 days after bout 1 (B1 + 27d) and two days after bout 2 (B2) (B2 + 2d). The arrows show a CD8^+^ T-cell as evidenced by CD8 immunoreactivity localized around a DAPI-positive nucleus. **(B)** Merged image reveals the CD8^+^ T-cell invading an apparently necrotic fiber. **(C)** CD8^+^ T-cell enumeration per muscle fiber before and 2 days following the first (B1) and second bout (B2) of damaging lengthening contractions (LC). ^*^ Indicates significant difference (One-way repeated measures ANOVA with Tukey's HSD, *p* < 0.05). Scale bar = 50 μm.

Our analysis of cytokines identified increases in few cytokines 48 h post-LC. Among the differentially altered cytokines, IP-10 was interesting, as we had previously found this cytokine to be increased 24 h following a single bout of LC (Hyldahl et al., 2014). IP-10 appears to be important in the activation and trafficking of T-cells to infected tissues (Khan et al., 2000; Dufour et al., 2002) and is found to be up-regulated in the muscle of individuals suffering from inflammatory myopathies accompanied by increased levels of cytotoxic T-cells (De Paepe et al., 2005). Guided by our finding of increased levels of IP-10 following B2, we assessed the presence of CD8^+^ T-cells in the muscle samples using an antibody against CD8 (Figures 4A,B). Overall, the occurrence of CD8^+^ T-cells in the muscle samples was infrequent relative to other cell types that have been identified in human skeletal muscle. Nevertheless, CD8^+^ T-cells per 100 muscle fibers increased following B2 (2.7 ± 2.8) relative to pre-B1 (0.5 ± 0.4) (*p* = 0.003) and post-B1 (1.0 ± 0.7) (*p* = 0.027) (Figure 4C). CD8^+^ cells were also found infiltrating what appeared to be regenerating myofibers, as evidenced by diminished and discontinuous dystrophin staining pattern (Figure 4B) and in spaces adjacent to what appeared to be vasculature (i.e., capillaries).

### Major histocompatibility complex class I (MHC-I)

**Figure 5:**
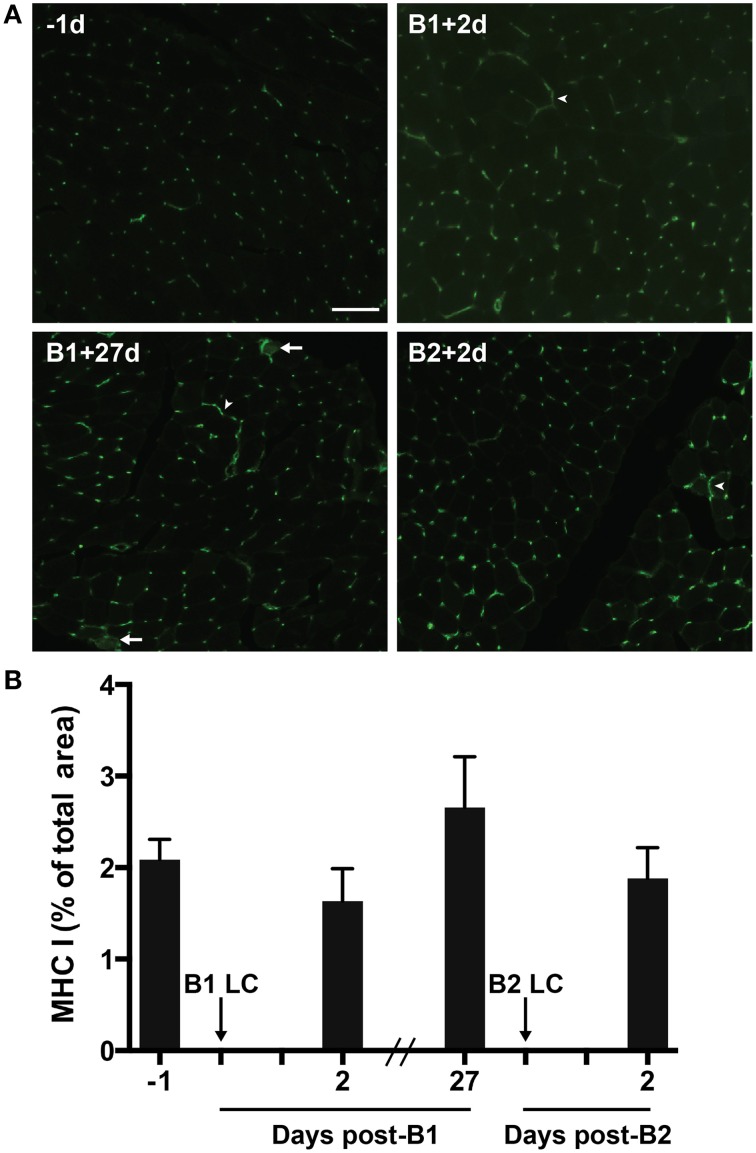
**Major histocompatibility complex class 1 (MHC-1) in skeletal muscle tissue**. **(A)** A representative florescent image of 8 μm thick muscle cross-sections stained for MHC-I. All four images were taken from muscle samples from the same subject 1 day before bout 1 (B1) of damaging lengthening contractions (LC) (−1d), 2 days after B1 (B1 + 2d), 27 days after bout 1 (B1 + 27d) and 2 days after bout 2 (B2) (B2 + 2d). Arrowheads show MHC-I positive sarcolemmal immunoreactivity. Arrows show MHC-I positive sarcoplasmic staining. **(B)** Percent of total muscle section positive for MHC-I before and after B1 and B2 of LC. No significant differences observed (One-way repeated measures ANOVA with Tukey's HSD, *p* < 0.05). Scale bar = 100 μm.

MHC-I proteins display antigens on the cell surface for CD8^+^ T-cell-mediated surveillance. MHC-I is not normally expressed by mature skeletal muscle cells. However, MHC-I expression can be greatly up-regulated in muscle suffering from inflammatory myopathies (Englund et al., 2001; Choi et al., 2009) and in response to extreme exercise (Marklund et al., 2013). Given the relationship between CD8^+^ T-cell and MHC-1, we measured MHC-I content via immunohistochemistry in the muscle to test the hypothesis that MHC-I content would increase in the muscle in parallel with the observed changes in T-cell content. Contrary to our hypothesis, we found no significant changes in MHC-I immunoreactivity (Figures 5A,B). MHC-I immunoreactivity was found consistently on capillaries and associated with interstitial mononuclear cells. MHC-I staining was also commonly found around the sarcolemma, though it was rarely found completely surrounding fibers (Figure 5A). In one subject (a high responder) evidence of sarcoplasmic MHC-I immunoreactivity was found (Figure 5A).

### Linear regression analyses

**Figure 6:**
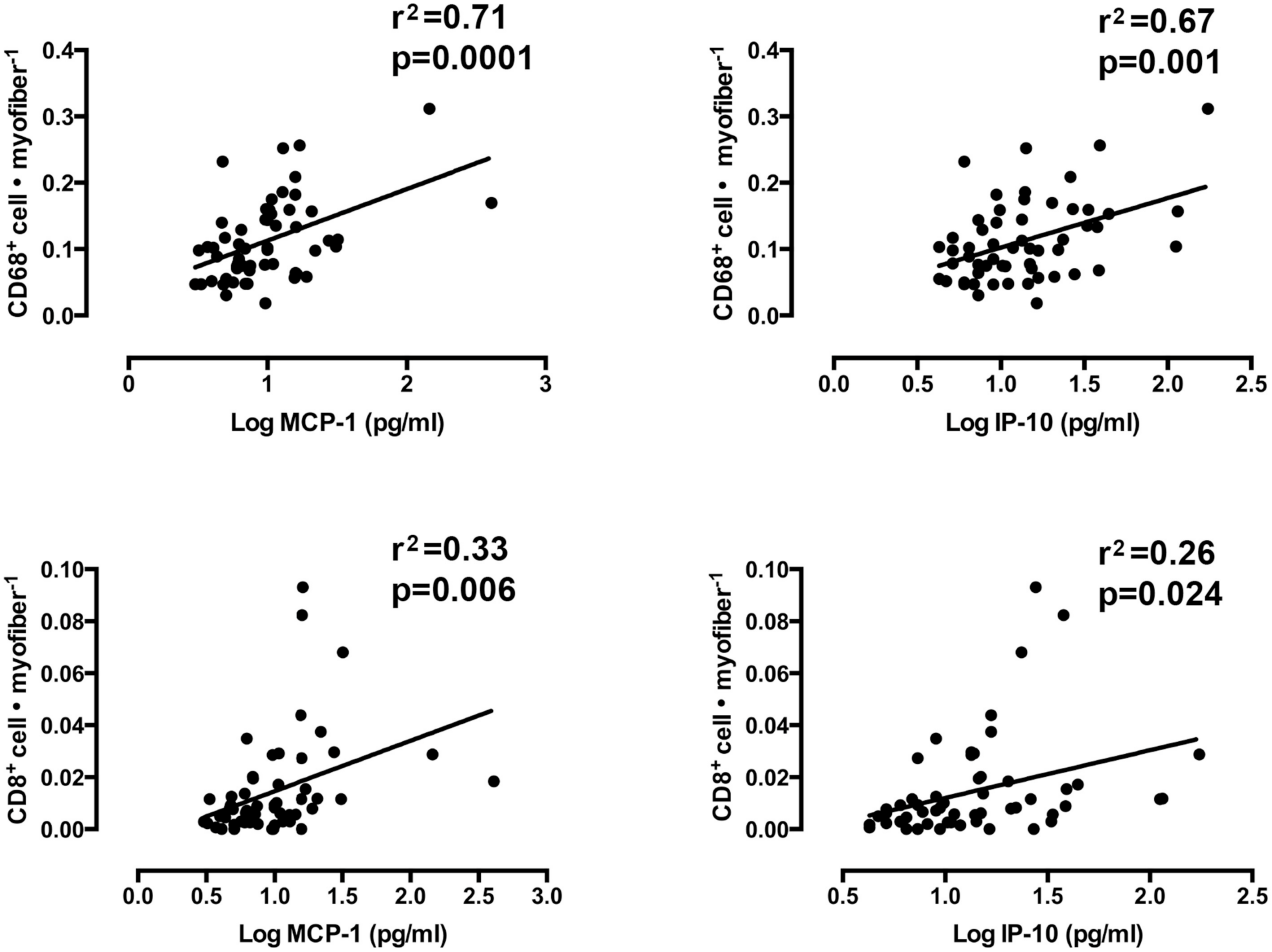
**The association between the number of muscle-infiltrating CD68^+^ macrophages and CD8^+^ T-cells and the protein content of monocyte chemoattractant protein 1 (MCP-1) and interferon gamma-inducible protein 10 (IP-10)**. (Mixed models linear regression, α = 0.05).

Mixed models linear regression analyses were used to determine if the content of the chemotactic cytokines, IP-10, and MCP-1 were related to the number of infiltrating CD8^+^ T-cells and CD68^+^ macrophages. Figure 6 shows graphical representations of each correlation. A strong relationship was found between log_e_ MCP-1 and CD68^+^ cells per muscle fiber (*r*^2^ = 0.71, slope = 0.029 ± 0.007, *p* < 0.0001). A similarly strong relationship was also observed between log_e_ IP-10 and CD68^+^ macrophages per muscle fiber (*r*^2^ = 0.67, slope = 0.027 ± 0.008, *p* = 0.001). Weaker, yet significant correlations were found between these cytokines and CD8^+^ T-cells. Log_e_ IP-10 vs. CD8^+^ T-cells per fiber yielded an *r*^2^ of 0.26 (slope = 0.007 ± 0.009, *p* = 0.024). MCP-1 vs. CD8^+^ T-cells per fiber yielded an *r*^2^ = 0.33 (slope = 0.008 ± 0.003, *p* = 0.006). No significant correlation between MHC-I immunoreactivity and CD8^+^ T-cell infiltration was found (*r*^2^ = 0.25, slope = −0.001±0.002 *p* = 0.71).

## Discussion

Inflammation following damaging exercise has been thought to cause further tissue damage mediated by muscle-invading leukocytes (Toumi and Best, 2003). A hypothesized mechanism of the RBE posits that inflammation following damaging exercise, and in turn secondary muscle damage, is blunted following a second bout of damaging exercise (McHugh et al., 1999). Overwhelmingly, the data presented here do not support the hypothesis of an attenuated inflammatory response following a second bout of LC. On the contrary, the data suggest an unaltered or slightly enhanced inflammatory response following a second bout of LC. The overall reduction in delayed onset muscle soreness (an established indicator of muscle damage, Clarkson and Hubal, 2002) in the days following B2 provides evidence of a RBE. Additionally, a previously published study that reported on the same group of subjects, showed reduced strength loss, and insignificant increases in serum creatine kinase activity following B2 (Hyldahl et al., 2015).

Surprisingly, only three cytokines showed significant changes among all 29 measured. Inflammatory cytokines MCP-1 and IP-10 increased after B2, while the anti-inflammatory cytokine IL-4 was reduced before and after B2. The increases in cytokine concentration also mirrored (and were positively correlated with) the changes in macrophage and T-cell infiltration, providing suggestive evidence for a chemotactic relationship between these cytokines and inflammatory cell populations in human muscle. Our observation that these events were only increased following B2 suggests that the initial bout of LC may have sensitized the muscle toward a greater, and therefore more persistent state of inflammation following the second bout of LC. In other words, the muscle seems to “remember” the damaging insult and is sensitized to initiate a more robust recruitment of immune cells in response to a repeated insult, reminiscent of the way the adaptive immune system responds to a repeated antigen exposure. Furthermore, muscle soreness is reduced concurrent with an increase in inflammation, indicating that a positive relationship between these variables is unlikely.

### Cytokines

Increases in MCP-1 following muscle damage have been widely reported in the animal and human literature, and its augmentation following damage appears to be necessary for healthy muscle regeneration (Warren et al., 2004; Shireman et al., 2007). Consistent with the findings of Hubal et al. (2008), we observed a significant increase in MCP-1 post-B2. However, this finding is in contrast with another study that showed an attenuation of MCP-1 gene expression following a repeated bout of damaging electrically stimulated isometric contractions (Mackey et al., 2011). Electrically stimulated contractions and voluntary eccentric contractions have been shown to cause distinct histological and biochemical responses (Crameri et al., 2007). Therefore, the reason for these disparate findings may be related to the differing modes of damage-induction. Furthermore, both of these studies measured MCP-1 at the transcript level, whereas we assessed MCP-1 protein content.

An interesting and novel finding that emerged from the present study is the increase in the classically pro-inflammatory cytokine IP-10 following B2. IP-10 is an important regulator of T-cell trafficking (Dufour et al., 2002; Wang et al., 2015). IP-10 is also involved in inflammation and immune responses in a host of tissues and conditions, yet little data are available regarding the role of IP-10 in skeletal muscle. IP-10 has been identified as a potential therapeutic target for inflammatory myopathies (Crescioli et al., 2012), but is also expressed in unperturbed healthy skeletal muscle (De Paepe et al., 2005). Recent work from our laboratory showed IP-10 to be up-regulated in muscle 24 h after a single bout of LC but not shortening contractions (Hyldahl et al., 2014). Like in our previous study, IP-10 concentrations were increased in response to damaging LC, albeit only after a repeated bout (Figure 2). Why significant increases were not seen post-B1 might be due to different sampling time points. In the present study, we took muscle samples 48 h after exercise whereas our previous investigation sampled muscle 24 h after exercise. Together, these studies suggest IP-10 may play a role in the damage and repair process of muscle adaptation to exercise. More work is needed to characterize the role IP-10 plays in these processes.

IL-4 is an important cytokine in driving the M1 to M2 phenotypic conversion (Villalta et al., 2009). Here we report a decrease in IL-4 protein concentration in muscle tissue from pre-B1 to post-B2 (Figure 2). This finding was unexpected because the present hypothesis predicted an attenuated inflammation response around B2, but the observed reduction in IL-4 around B2 is suggestive of an increased pro-inflammatory state. These data suggest that the increased number of CD68^+^ macrophages after B2 (Figure 3B) may have embodied a more pro-inflammatory phenotype. However, TNF-alpha and IFN-gamma, both promoters of the M1 phenotype (Villalta et al., 2009) were not present in the muscle samples at this time point in detectible quantities (Table 1). This suggests that the macrophages present post-B2 likely did not display a highly polarized phenotype at either end of the M1/M2 spectrum. Given that macrophages were significantly elevated only at post-B2 when evidence of muscle damage was reduced suggests that these cells do not aggravate the symptoms (i.e., soreness, force loss, and membrane permeability) of exercise-induced damage in healthy human skeletal muscle. This interpretation is consistent with data from Tidball et al. (1999), which showed that the loss of sarcolemmal integrity in response to muscle overload was not different in the presence or absence of macrophages. Likewise, another study by the same group showed that macrophages promote muscle repair and regeneration in response to overload-induced damage (Tidball and Wehling-Henricks, 2007).

### Inflammatory cell infiltration

Consistent with others (Stupka et al., 2001; Mackey et al., 2011) we observed a significant increase in macrophage content in the muscle following damaging exercise, though we are the first, to our knowledge, to report that macrophages accumulate in greater numbers following a second exposure to LC. Another novel finding of the present study is our observation of increased muscle T-cell content after a second bout of LC (Figure 4C). T-cells are known to play a role in inflammatory myopathies (Choi et al., 2009; Crescioli et al., 2012), muscular dystrophies (Spencer et al., 2001; Choi et al., 2009; Madaro and Bouche, 2014), and obesity-related muscle fat accumulation and insulin resistance (Khan et al., 2015). Moreover, the presence of T-cells in muscle tissue has been thought to be specific to muscle afflicted with chronic inflammatory pathologies (Madaro and Bouche, 2014). However, one study has demonstrated a significant increase in muscle T-cell content following a 24-h ultra endurance bout (Marklund et al., 2013). The significant increase in T-cells observed after B2 in the present study suggests that these cells may play a role in the adaptive and regenerative processes of healthy human skeletal muscle to damaging exercise. Indeed, recent animal studies show that T-cells play a role in muscle regeneration by chemokine and cytokine secretion (Zhang et al., 2014; Fu et al., 2015). A study by Malm et al. (2000) measured no changes in infiltrating CD8^+^ T-cells in healthy muscle tissue in response to a single bout of damaging eccentric cycling exercise. The authors concluded that T-cells do not infiltrate healthy skeletal muscle. Consistent with this study we also did not detect a significant change in T-cell content after a single bout of damaging exercise. However, we show that CD8^+^ T-cells do infiltrate healthy muscle, but only do so in appreciable quantities after a repeated bout of damaging exercise. It may be that an initial bout of exercise primes the muscle to more effectively recruit T-cells that become manifest at a subsequent bout.

Because no significant changes in MHC-1 content was observed, the priming effect may not be mediated by increased muscle MHC-I expression as we had suspected. However, it is possible that damaged muscle may display peptide sequences on MHC-I proteins that are indicative of muscle damage (damage-specific epitope), thereby sensitizing T-cells to respond to muscle damage by recognizing muscle damage-specific auto antigens displayed on MHC-I without detectible changes in total MHC-I content. This speculation provides a hypothesized mechanism of the observation that CD8^+^ T-cells increased only after B2. Another possible explanation is that T-cells were recruited to the muscle slowly after B1, were insignificantly increased at the 27 day time point (Figure 4C), and the higher numbers of T-cells that accumulated in the muscle pre-B2 allowed for a more rapid T-cell expansion by proliferation, recruitment of more T-cells, or both in response to LC of B2. Consistent with this hypothesis, the slightly increased MHC-I content at pre-B2 (statistically insignificant, Figure 5B) may play a role in sensitizing T-cells for heightened responsiveness post-B2. It may be that slightly increased MHC-I expression at this time point provided low-affinity sub-threshold interactions with MHC-bound non-damage-indicative peptides and the T-cells present in the tissue. T-cells are known to interact in this way with self-peptides (a phenomenon known as tonic stimulation). Tonic stimulation is important for T-cell homeostasis and may render T-cells more readily activatable upon foreign antigen exposure (Stefanová et al., 2002). Perhaps the higher, yet statistically insignificant increase in MHC-I at pre-B2 may have provided more tonic stimulation and heightened the T-cell response post-B2 when, perhaps, a damage-specific epitope was displayed on MHC-I.

Interestingly, CD8^+^ T-cells seemed to aggregate around and invade necrotic muscle fibers (necrotic fibers were very rarely observed). A representative image of this phenomenon is shown in Figure 4B. This may suggest that T-cells promote muscle damage by inducing muscle fiber death, or may serve a phagocytic role following fiber damage. The interpretation that CD8^+^ T-cells play a deleterious roll in muscle by promoting muscle fiber damage is supported by the observation that depletion of these cells significantly reduced pathologic symptoms in murine models of Duchenne muscular dystrophy (Spencer et al., 2001) and dysferlinopathy (Farini et al., 2012). However, given the present observation that CD8^+^ T-cells are only significantly increased after B2 (Figure 4C) when evidence of muscle damage was reduced (Figures 1A,B), suggests that these cells do not exacerbate exercise-induced damage in non-dystrophic human muscle. Rather, the present data are more consistent with a recent rodent study, showing that CD8^+^ T-cells facilitate muscle repair via MCP-1 chemokine expression, thus assisting in the recruitment of tissue-repairing macrophages (Zhang et al., 2014).

### Study limitations

Limitations of the present study include (1) the potential inflammation promoting effect of the biopsy procedure, and (2) the single 48 h sampling time point following exercise. Though the biopsies were taken 3 days apart, studies have shown that multiple muscle biopsies taken from the same muscle in close proximity are capable of promoting changes in inflammatory cytokine transcript level in subsequent biopsy samples (taken 2 days apart) independent of exercise (Van Thienen et al., 2014). On the contrary, other studies have shown markers of damage and inflammatory events in only the exercised, but not the rested limbs (Mackey et al., 2008; Lauritzen et al., 2009). To reduce the effects of the previous biopsy procedure on the measured inflammatory markers, the post-B2 biopsy was always taken proximal to the previous biopsy site with the needle angled away from the previous biopsy site. This practice has been shown to minimize inflammation-related artifact from a previous biopsy procedure (Van Thienen et al., 2014). Moreover, we examined cytokine concentrations at the protein level, which appears to be far less affected by the biopsy procedure compared to mRNA expression (Van Thienen et al., 2014). It is also not likely that the biopsy performed prior to B2 negatively impacted LC done on the following day. A previously published paper that used the same subjects presently reported showed that the amount of work done in B1 (42.3 ± 9 kJ) was not statistically different from, that done in B2 (43.3 ± 17 kJ) (*p* = 0.42) (Hyldahl et al., 2015).

In order to minimize the total number of biopsies, yet assess inflammation markers during the period of peak muscle soreness and strength loss, we chose to sample 48 h following each bout of LC. It is possible that changes in some of the inflammatory markers were missed if they occurred earlier or later than 48 h after exercise. It is possible that the magnitudes of the inflammatory response at B1 and B2 were not different early after exercise prior to the 48 h time point, and that inflammation post-B2 simply persisted longer.

## Conclusions

The data presented here do not support the hypothesis that blunted inflammation following a repeated bout of LC explains the attenuated symptoms that accompany the RBE. Rather, we observed increased inflammation in muscle tissue concomitant with reduced evidence of muscle damage following B2. Moreover, the inflammatory environment in the muscle following B2 seemed to be optimized to facilitate recruitment of immune cells as evidenced by increased levels of chemokines MCP-1 and IP-10. Indeed, muscle-infiltrating CD68^+^ macrophages as well as CD8^+^ T-cells were also elevated following B2 and were significantly correlated with the content of both chemokines. Collectively, the data suggests that in response to an initial bout of damaging exercise the muscle becomes more effective at recruiting immune cells following a repeated bout of LCs and that these cells may facilitate accelerated repair to the muscle and thereby contribute to the RBE. This study also provides the first observation that CD8^+^ T-cells may be involved in the adaptive processes of healthy human skeletal muscle in response to a repeated bout of damaging exercise. Lastly, the increases in CD8^+^ T-cells were seen in the absence of significant increases in MHC-I.

## Author contributions

MD was responsible for conducting experiments, analyzing data, writing, and editing the manuscript. AG and KE ran experiments and helped write and edit the manuscript. DE helped with the statistical modeling and analysis. WN ran experiments and helped edit the manuscript. AP helped design the study, collected muscle biopsy samples and helped edit the manuscript. RH collected data, ran experiments, analyzed data, helped write, and edit the manuscript.

### Conflict of interest statement

The authors declare that the research was conducted in the absence of any commercial or financial relationships that could be construed as a potential conflict of interest.
